# Behavioral Differences of Laying Hens with Fractured Keel Bones within Furnished Cages

**DOI:** 10.3389/fvets.2016.00042

**Published:** 2016-05-31

**Authors:** Teresa M. Casey-Trott, Tina M. Widowski

**Affiliations:** ^1^Animal Biosciences, University of Guelph, Guelph, ON, Canada

**Keywords:** behavioral change, resting location, pain, keel bone fracture, furnished cage, causation

## Abstract

High prevalence of keel bone fractures in laying hens is reported in all housing systems. Keel fractures have been associated with pain and restricted mobility in hens in loose housing. The objective was to determine whether keel fractures were associated with activity of hens in furnished cages. Thirty-six pairs of LSL-Lite hens (72 weeks) were enrolled in the study. One hen with a fractured keel and one hen without were identified by palpation in each of 36 groups of hens housed in either 30- or 60-bird cages stocked at 750 cm^2^/hen. Behavioral activity of each hen was recorded by four observers blind to keel status using focal animal sampling for 10 min within a 2-h period in the morning (08:00–10:00), afternoon (12:00–14:00), and evening (17:00–19:00). All hens were observed during each of the three sample periods for 3 days totaling 90 min, and individual hen data were summed for analysis. Hens were euthanized 48 h after final observations, dissected, and classified by keel status: *F*_0_ (no fracture, *N* = 24), *F*_1_ (single fracture, *N* = 17), and *F*_2_ (multiple fractures, *N* = 31). The percentages of time hens performed each behavior were analyzed using a mixed procedure in SAS with fracture severity, body weight, cage size, rearing environment, and tier in the model. Fracture severity affected the duration of perching (*P* = 0.04) and standing (*P* = 0.001), bout length of standing (*P* < 0.0001), and location (floor vs. perch) of resting behaviors (*P* = 0.01). *F*_2_ hens perched longer than *F*_0_ hens, 20.0 ± 2.9 and 11.6 ± 3.2%. *F*_2_ hens spent less time standing, 15.2 ± 1.5%, than *F*_0_ and *F*_1_ hens, 20.7 ± 1.6 and 21.6 ± 1.8%. *F*_2_ hens had shorter standing bouts (22.0 ± 4.2 s) than both *F*_0_ and *F*_1_ hens, 33.1 ± 4.3 and 27.4 ± 4.4 s. Non-fractured hens spent 80.0 ± 6.9% of total resting time on the floor, whereas *F*_1_ and *F*_2_ hens spent 56.9 ± 12.4 and 51.5 ± 7.7% resting on the floor. Behavioral differences reported here provide insight into possible causes of keel damage, or alternatively, indicate a coping strategy used to offset pain or restricted mobility caused by keel fractures.

## Introduction

The keel bone is known to be a site of frequent fractures during the production life of laying hens with incidence rates ranging from 5% to over 85% (1–3). Although the prevalence is typically lowest in conventional cages (4), birds in all types of housing systems are susceptible to keel fractures. The high occurrence of these fractures is alarming, as the welfare of the bird is potentially compromised by this reportedly painful condition (5).

Observing behavior provides insight into the internal state of an animal, and behavioral measurements have been used to assess pain in animals (6) including poultry (7–9). Understanding the behavioral changes typically associated with pain provide an opportunity for researchers, producers, or welfare auditors to assess the current state of an animal within its given housing situation. In birds, evidence of decreased spontaneous activity (7), reduced mobility (9), and reduced latency-to-lie (9, 10) are reported in association with lameness, and changes in the amount of standing and sitting behaviors are influenced by pain as levels of these activities were improved or restored with analgesic treatments (8, 11).

In addition, understanding where inactive behaviors occur within the housing system also provides useful information regarding resting and coping strategies. Broiler chickens adjust their sleeping location to reduce disturbances by resting near walls when housed at high densities (12), and similar strategies are used by laying hens by resting on perches to reduce disturbances (13) and social interactions (14). In relation to the keel bone in particular, resting location has the potential to have a significant impact. Provision of perches has been shown to increase keel deformities and fractures (15), and even the perch material itself can affect the prevalence and severity of keel damage (16, 17). Where the hens rest within the cage may provide insight into the coping strategy of hens with keel damage or offer information on how the keel damage occurred in the first place.

The keel bone, in particular, is a critical bone to the avian skeleton, as it is a structure responsible for the ability of flight in birds and plays an important role in driving the respiratory process in avian species (18–20). The keel serves as an anchor for attachment of flight muscles necessary for wing flapping and short bursts of flight required by fowl species to reach roosting sites and for escape strategies to avoid predators (18). Abdominal muscles also attach to the keel allowing for ventral and dorsal oscillations of the keel to aid in the filling and emptying of the air sacs during inhalation and exhalation in birds (19, 20). This mechanism is essential for adequate ventilation of the lungs, as birds lack a muscular diaphragm and respiratory processes of birds operate with the lung volume remaining static in contrast to the dramatic lung volume changes required for mammalian respiration (18).

Since the keel bone plays an important role in avian behaviors such as flight and wing flapping, as well as vital processes such as respiration, it is possible that damage to the keel bone limits daily functioning of the bird and subsequently alters behavior and activity levels throughout the day. Nasr et al. (21) reported that daily activity of hens group housed in floor pens was altered by the presence of keel fractures. Hens with keel fractures spent more time sleeping on the floor and less frequently accessed perches of various heights compared to hens without keel fractures. Individual hen testing also demonstrated that hens without keel fractures completed walkway tests faster and had a shorter latency to fly down from perches compared to hens with keel fractures (5, 21). While these tests provide evidence that hens with keel fractures have altered mobility, they offer minimal insight into the behavioral differences of hens with keel damage in a furnished cage environment using commercially available equipment and industry standard stocking density.

The objective of this study was to quantify general behavior differences between hens with fractured keels and hens without fractured keels housed in furnished cages. Determining where injured birds spend their time resting within the cage as well as which behaviors may be inhibited or amplified in hens with keel damage can provide information on how to single out hens with keel injuries and has the potential to add to the discussion of the causes, consequences, and welfare implications of how keel damage occurs in different types of housing systems. We hypothesized that behavior would differ between hens with or without keel bone damage, especially in regard to inactivity, namely, sitting, standing, and sleeping, and that hens with keel damage would likely spend more time perching.

## Materials and Methods

### Animal Housing

Animal use was approved by the University of Guelph Animal Care Committee (Animal Utilization Protocol #1947). Three consecutive flocks of 540 Lohmann-selected Leghorn Lite laying hens were reared from 1 day of age to 73 weeks of age at the University of Guelph Arkell Poultry Research Station. As part of another concurrent experiment, half of the hens were reared in conventional cages (*N* = 270) and half were reared in an aviary system (*N* = 270). At 16 weeks of age, hens from both rearing systems were placed into two rooms holding 12 Farmer Automatic Enrichable (Furnished) Cages (Clark Ag Systems, ON, Canada) each, with one rearing treatment placed into each cage. Each room contained 6 large (41,296 cm^2^; 60 hens) and 6 small (20,880 cm^2^; 30 hens) cages, both providing 750 cm^2^/hen space allowance. Placement of rearing treatments was balanced for cage size. Each bank of six cages had three tier levels with one large and one small cage on each tier. The same rearing and adult rooms were used for each consecutive flock.

Hens were fed a commercial layer crumbled pellet diet with automatic feed chains running every 3 h commencing at the start of a 14-h light period from 0500 to 1900 hours with a 15-min sunrise and sunset starting at 500 and 1845. The light intensity varied among tiers, with the highest intensity recorded on the top tiers measuring 10–15 lux and the lowest intensity at the bottom tiers measuring 4–5 lux. Each furnished cage provided a curtained nest area proportional to cage size, 10 cm high perches running parallel throughout middle area, and a smooth plastic scratch area. Nipple drinkers with cups were located above the auger down the middle of the cage. The feed troughs were located on both outer sides of the cages. All rooms were sealed and entirely lit with artificial light with no natural, external light sources present. Beak trimming was performed at the hatchery at 1 day of age using infrared treatment.

The mean laying rate at 70 weeks was 93.2 ± 1.0% SE for flock 1, 90.6 ± 1.2% SE for flock 2, and 93.2 ± 0.6% SE for flock 3. The mean flock mortality was 3.3 ± 0.9% SE for flock 1, 4.7 ± 1.0% SE for flock 2, and 5.5 ± 1.1% SE for flock 3. All mortalities were sent for postmortem analysis, and there were no outbreaks of disease, feather pecking, or cannibalism throughout the duration of the study. Only hens of normal body weight and free from moderate to severe foot damage were included in the treatment selection process.

### Treatment Selection

A set of 12 pairs of LSL-Lite hens were selected for inclusion in this study at 72 weeks of age from each of three consecutive flocks for a total of 72 hens. Selection for enrollment in the study was based on the keel status of each individual bird determined by palpation. Lights were dimmed for ease of handling, and hens were caught randomly from within each furnished cage until one hen with a normal, non-fractured keel and one hen with a fractured keel was found. A keel was considered non-fractured if it followed a normal, straight 180° line without the presence of any sharp bends or periosteal scars or callus indicative of a healing fracture. A keel was classified as fractured if there was the presence of a sharp bend or deviation from the 180° line accompanied by one or more than one periosteal scar or callus (22). Three days prior to the observation period, each fractured and non-fractured hen was marked with colored, numbered livestock ID tags (Allflex, QC, Canada), with one tag placed in each wing web with an ATag One Piece Applicator (Allflex, QC, Canada) to allow for visual identification within the cage.

### Behavioral Observations

Four trained observers, blinded to keel status, recorded behavioral activity following a specified ethogram (Table 1) with the number of observations balanced between rooms and cages. Reliability among all four observers was determined by using Kendall’s Coefficient of Concordance (*W*) to rank the behaviors recorded by each observer. Agreement among observers was acceptable (*W* = 0.725; X^2^ 139 = 11.6; *P* = 0.0206). According to Martin and Bateson (23), inter-observer reliability scores above 0.70 are considered to be acceptable, especially when multiple observers are used. The observers were balanced across keel status category, with 34.5–36.7% of each observer’s total observations in the *F*_0_ category, 25.1–30.3% of each observer’s total observations in the *F*_1_ category, and 32.9–40.2% of each observer’s total observations in the *F*_2_ category. *Each* hen was continuously recorded using a handheld machine (Psion Workabout Pro3, Motorola Solutions, Schaumburg, IL, USA) to collect data using Pocket Observer software (Observer XT, Noldus Information Technology, Wageningen, The Netherlands) for a period of focal animal sampling of 10 min within a sample period of 2 h in the morning (08:30–10:30), afternoon (12:30–14:30), and evening (17:00–19:00) repeated for 3 days. Nighttime resting location could not be recorded since the majority of hens huddled in a group on the floor of the cage and the individual hen ID tags were not visible. To reduce the confounding effect of behaviors related to nesting, observations began after the completion of the peak morning laying period for each flock, as determined by research previously completed on-site (24).

**Table 1:** **Ethogram used for behavior observations**.

Behavior	Description
**General activity**	
Forage	Pecking or scratching at the floor of the cage with head below rump [adapted from Klein et al. (25)]. Can include scratching motion with legs but does not include feather pulling/pecking on other birds
Eat	Head in the feed trough or completely through the cage over the feeder. Can include standing breaks of ≤5 s followed by resumption of behavior
Drink	Repeated pecks at nipple drinker followed by swallowing. Can include standing breaks of ≤5 s, with beak still within the plane of the drinker, followed by resumption of drinking behavior
Preen	A hen uses her beak to clean wing and body feathers. Related behaviors include head scratching, wing stretching, feather ruffling, and/or feather erection
Walk	Moving more than three paces in one direction, head erect
Stand	Hen standing on feet, legs extended, no movement of the body but with eyes open [adapted from Webster and Hurnik (26)]. Head in either erect or relaxed posture
Sit	Hen’s body is flush with the bottom of the cage, wings tucked, and head either erect or in relaxed posture. Eyes are open
Sleep	Hen in a relaxed posture, either sitting or standing, with eyes closed. Head may be tucked [adapted from Blokhuis (27)]
Dust bathe	A hen performs vertical wing shakes on the wire, bill raking, circular foot motions. Includes sham dustbathing. Hen may pull feed from feeder to use as substrate. Can be social or individual [adapted from Scholz et al. (28)]
Perch	A hen has two feet on a perch (or feed auger) for more than 3 s (i.e., not stepping over the perch)

The time of observation for each cage was shifted everyday by 40 min within the 2-h period to ensure observation of each hen occurred at a different time point within the 2-h period on all 3 days. The duration of general activities (forage, eat, drink, preen, sit, stand, walk, sleep, dust bathe, and perch) described by the ethogram were recorded and the data for each hen over all 3 days was summed for analysis. All behaviors, except perching, were recorded as mutually exclusive state behaviors in that initiation of one behavior terminated the recording of the previous behavior. Simultaneous location (nest box, cage middle, scratch area, and cage front) recording was recorded for all behaviors. Occurrence of perching was recorded in conjunction with any behaviors occurring while the hen was located on the perch allowing for a description of specific activities occurring on the perches.

Within 48 h following the final observations, hens were killed by cervical dislocation, dissected, and re-classified by keel status at dissection: non-fractured: *F*_0_ (no fracture and no deviation from 180°, *N* = 24); minor fracture: *F*_1_ (single, “greenstick” fracture at the caudal tip of the keel without any deviation from 180°, *N* = 17); severe fracture: *F*_2_ [multiple fractures (including at least one complete fracture) with deviation from 180°, *N* = 31].

### Statistical Analyses

For all statistical procedures, only the true damage status of the keel, as determined by dissection, was used in the analyses. Although the sensitivity and specificity of the identification of keel status by palpation are high (3, 22), reclassification of keel status at dissection is commonly used in keel bone research (5, 21) to ensure accurate treatment allocation. Fracture severity classified dissected keels as non-fractured (*F*_0_), minimally fractured (*F*_1_), or severely fractured (*F*_2_), as described above.

### Analysis of General Activity

The total duration of each behavior within the ethogram for each individual hen was summed and used to create a proportion of total time out of the 90-min that each hen was observed and then used for analysis in SAS 9.4 (SAS Institute Inc., Cary, NC, USA). Each behavior was assessed in a mixed model analysis of variance with fracture severity (*F*_0_, *F*_1_, and *F*_2_), body weight, rearing environment (conventional cage and aviary), cage size (large and small), and tier (top, middle, and bottom) in the model as fixed effects. Flock number and room were included as random effects to account for any flock and room variation. Interactions were initially included in the model, but were subsequently removed due to lack of significance. All data were tested for normality and normality of residuals and the proportion of sleeping behavior required arc sin square-root transformation.

The mean bout lengths and bout frequencies for sitting, standing, and sleeping were also analyzed. A mixed model procedure with fracture severity (*F*_0_, *F*_1_, and *F*_2_), body weight, rearing environment (conventional cage and aviary), cage size (large and small), and tier (top, middle, and bottom) in the model as fixed effects. Flock number and room were included as random effects to account for any flock or room variation. Interactions were initially included in the model, but were subsequently removed due to lack of significance. Square-root transformation was used to achieve normality for the bout length and bout frequency for sleep.

### Analysis of Resting Behavior Location

With the intention of assessing differences in resting location between hens with or without keel damage, only hens that exhibited resting behavior could be assigned a resting location and subsequently be included in the analysis of resting location. A category of resting behavior was created by combining the duration of all sitting and sleeping behavior for an individual hen. Hens that rested for at least 10% of their total observation period, with resting bouts occurring within a minimum of three of the nine 10-min observation periods were included in this data set. A total of 48 hens met this criteria (*N* = *F*_0_:16, *F*_1_:9, and *F*_2_:23). The proportion of time resting on either the perch or the floor of the cage was then assessed in a mixed model procedure with fracture severity (*F*_0_, *F*_1_, and *F*_2_), body weight, rearing environment (conventional cage and aviary), cage size (large and small), and tier (top, middle, and bottom) in the model as fixed effects. Flock number and room were included as random effects to account for any flock or room variation. Interactions were initially included in the model, but were subsequently removed due to lack of significance. The proportion of time resting on the perch and the proportion of time resting on the floor were transformed using arc sin square-root.

## Results

### General Activity

Keel fracture severity had an effect on the percentage of time perching (*F*_2,61_ = 3.30, *P* = 0.0436; Table 2). Hens with a keel status of *F*_2_ perched for a greater percentage of time (20.0 ± 2.9% SE) than both *F*_0_ and *F*_1_ hens, 11.6 ± 3.2 and 13.9 ± 3.6% SE, respectively. Rearing environment (*F*_1,61_ = 4.26, *P* = 0.0432) and cage size (*F*_1,61_ = 5.34, *P* = 0.0243) also affected perching. Aviary reared hens spent more time on the perches that conventionally reared hens, 18.2 ± 2.8 and 12.7 ± 2.8% SE. Hens in small cages spent more time perching (18.6 ± 2.8% SE) compared to hens in large cages (11.7 ± 2.9% SE). There was no effect of body weight (*F*_1,61_ = 0.43, *P* = 0.5130) or tier (*F*_2,61_ = 1.14, *P* = 0.3256) on the percentage of time perching.

**Table 2:** **Description of each behavior as a percentage of the total 90-min observation period**.

	*F*_0_ *N* = 24 %	*F*_1_ *N* = 17 %	*F*_2_ *N* = 31 %	*P*-value
Forage	7.2 ± 2.7	7.7 ± 2.8	9.3 ± 2.6	0.4034
Eat	23.7 ± 3.1	26.2 ± 3.5	24.2 ± 2.9	0.7973
Sit	10.9 ± 2.0	12.8 ± 2.3	13.8 ± 1.8	0.4744
Stand	20.7 ± 1.6^a^	21.6 ± 1.8^a^	15.2 ± 1.5^b^	**0.0011**
Walk	7.4 ± 0.8	8.4 ± 0.9	8.4 ± 0.7	0.1536
Drink	6.9 ± 0.7	7.4 ± 0.8	6.7 ± 0.6	0.7766
Preen	7.5 ± 1.1	10.4 ± 1.4	9.8 ± 1.0	0.2116
Sleep	9.1 ± 2.1	4.4 ± 0.9	9.9 ± 1.8	0.2423
Dust bathe	1.6 ± 0.05	1.1 ± 0.04	1.1 ± 0.03	0.9559
Other	5.0 ± 0.6	0	1.6 ± 0.4	–
Perch	11.6 ± 3.2^a^	13.9 ± 3.6^a^	20.0 ± 2.9^b^	**0.0436**

*All behaviors were mutually exclusive, except for perching which was recorded in conjunction with any behavior occurring while the hen was on the perch*.

*^a,b^ Differences in designate statistical significant difference of P < 0.05*.

*Bold indicates statistical significance*.

Keel fracture severity also had an effect on the percentage of time standing (*F*_2,61_ = 7.65, *P* = 0.0011; Table 2). Hens with an *F*_2_ keel spent less time standing (15.2 ± 1.5% SE) than both *F*_0_ and *F*_1_ hens, 20.7 ± 1.6 and 21.6 ± 1.8% SE, respectively. There was no effect of body weight (*F*_1,61_ = 1.45, *P* = 0.2329), rearing environment (*F*_1,61_ = 2.46, *P* = 0.1217), cage size (*F*_1,61_ = 1.93, *P* = 0.1700), or tier (*F*_2,61_ = 0.43, *P* = 0.6524) on the percentage of time standing. The effect of keel fracture severity on all other behaviors can be found in Table 2.

**Figure 1:**
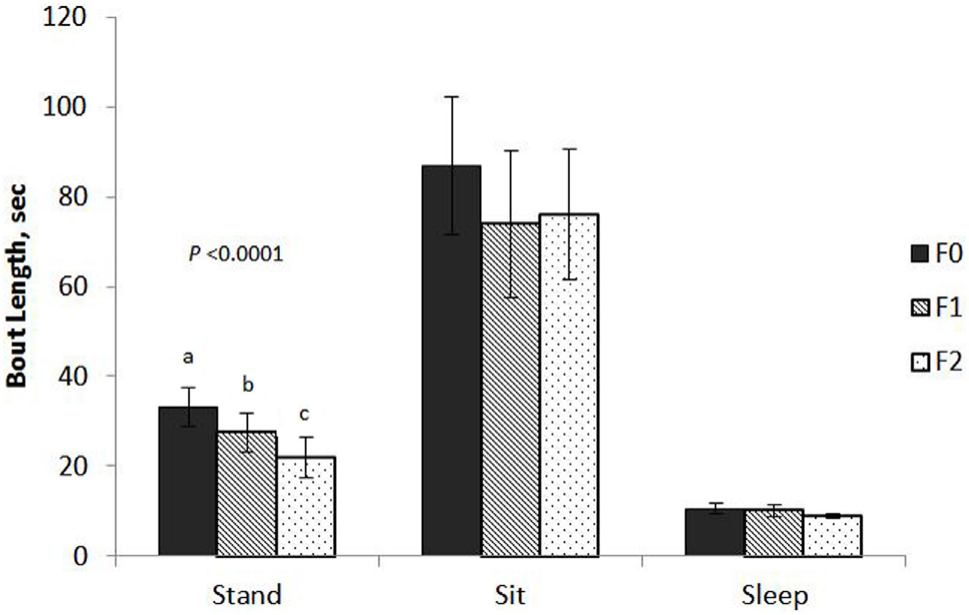
**Mean bout length of sitting, standing, and sleeping behaviors for hens with varied keel status**. Fracture severity is described as follows: non-fractured: *F*_0_ (no fracture and no deviation from 180°, *N* = 24); minor fracture: *F*_1_ (single, “greenstick” fracture at the caudal tip of the keel without any deviation from 180°, *N* = 17); severe fracture: *F*_2_ [multiple fractures (including at least one complete fracture) with deviation from 180°, *N* = 31].

Keel fracture severity had an effect on the mean bout length of standing (*F*_2,61_ = 11.88, *P* < 0.0001; Figure 1) with *F*_2_ hens standing for shorter bouts (22.0 ± 4.2 s SE) that both *F*_1_ (27.4 ± 4.4 s SE) and *F*_0_ (33.1 ± 4.3 s SE) hens. Cage size also affected standing bout (*F*_1,61_ = 8.09, *P* = 0.0060) with hens in small cages exhibiting a longer bout length (30.4 ± 4.2 s SE) compared to hens in large cages (24.7 ± 4.2 s SE). Keel fracture severity did not affect bout length for sitting (*F*_2,61_ = 0.46, *P* = 0.6347) or sleeping (*F*_2,55_ = 1.23, *P* = 0.3012).

The effect of fracture severity on the frequency of sitting bouts approached significance (*F*_2,61_ = 2.84, *P* = 0.0659) with *F*_2_ hens having a greater mean bout frequency (10.5 ± 1.2 SE) compared to *F*_1_ (8.2 ± 1.5 SE) and *F*_0_ (7.4 ± 1.3 SE). The number of bouts for standing and sleeping were not significant, *F*_2,61_ = 2.27, *P* = 0.1121 and *F*_2,55_ = 2.23, *P* = 0.1165.

Body weight had an inverse relationship with the percentage of time foraging (*F*_1,61_ = 5.89, *P* = 0.0182) and walking (*F*_1,61_ = 10.76, *P* = 0.0017); however, body weight accounted for only a small degree of variation based on the low *R^*2*^* values (0.0729 and 0.1619). Tier also had an effect on the percentage of time foraging (*F*_2,61_ = 3.51, *P* = 0.0359) with hens on the top tier spending more time foraging (10.6 ± 2.7% SE) compared to the bottom tier (6.4 ± 2.6% SE). The middle tier did not differ from either the top of bottom tier in terms of time foraging (7.2 ± 2.6% SE).

### Resting Behavior Location

Fracture severity and cage size had an effect on the location of resting behavior. The percentage of time resting on the floor of the cage was significantly greater for *F*_0_ hens, 80.0 ± 6.9% SE compared to *F*_1_ and *F*_2_ hens, 56.9 ± 12.4 and 51.5 ± 7.7% SE (Table 3; *F*_2,37_ = 4.63, *P* = 0.0161), and subsequently *F*_1_ and *F*_2_ hens spent a greater percentage of time resting on perches compared to *F*_0_ hens (Table 3). A larger percentage of hens in large cages rested on the floor of the cage (79.5 ± 8.7% SE) compared to hens in small cages (47.5 ± 8.4% SE; *F*_1,37_ = 11.52, *P* = 0.0015), and subsequently small cages had a larger percentage of hens resting on the perches than hens in large cages.

**Table 3:** **Association between fracture severity and the percentage of time resting on the floor vs. perch**.

	*F*_0_ *N* = 16 %	*F*_1_ *N* = 9 %	*F*_2_ *N* = 23 %	*P*-value
Rest on perch	20.2. ± 6.9^a^	42.5 ± 12.5^b^	48.1 ± 7.8^b^	0.0114
Rest on floor	80.0 ± 6.9^a^	56.9 ± 12.4^b^	51.5 ± 7.7^b^	0.0161

*^a,b^ Differences in designate statistical significant difference of P < 0.05*.

## Discussion

The primary objective of this study was to assess whether hens with keel bone damage behave differently or rest in different locations than hens without keel bone damage, and it is the first to consider this relationship within a commercially available, furnished cage setting. Although we predicted that differences in all inactive behaviors would be associated with keel status, only standing behavior was significantly different between fractured and non-fractured hens. Differences in sitting and sleeping behaviors were not detected. Our prediction that hens with keel fractures would spend more time perching and resting on the perches than hens without keel damage was correct. This prediction was based on several studies that suggest the perches may cause keel bone damage (2, 3, 15, 29, 30).

In contrast to Nasr et al. (21) who reported that hens with keel fractures spent more time sleeping on the floor and less time up on the perches, our results indicate the opposite. This is likely due to the difference in housing design, floor pen vs. furnished cage, which allow for dramatically different perch heights. Unlike the perches used by Nasr et al. (21), which ranged from 50 to 150 cm off the ground, the furnished cage perch is only 10 cm off the cage floor and requires no flight or jumping for access. As it is suggested that keel fractures are painful and restrict mobility, especially in regard to flight, the theory that pain or restricted flight mobility deters the hen from perching applies to Nasr et al. (21) and likely other non-cage, perch systems; however, it is not applicable to the furnished cage. The differences in results reported here in comparison to Nasr et al. (21) highlight the importance of considering how housing environment can alter the expression of pain behavior.

The current study details behavioral differences specific to hens housed in furnished cage systems. The significant differences in perch use and standing behavior among hens with varying degrees of keel bone damage provides evidence of a potential causal link between certain behaviors that may leave the hen more susceptible to keel damage, or alternatively offers insight into pain or physiological function-related changes that result from damage to the keel bone.

Several authors have suggested that although perches satisfy a motivated behavior, they actually cause more keel bone damage (2, 3, 15, 29, 30) most often in the form of crashes or falls. However, even in the relatively low-impact environment provided by furnished cages, it seems that there is still a relationship between perches and keel damage. Although impact injuries are most often discussed regarding perches and keel damage, the type of perch material, shape, peak force, and contact area between the keel and the perch surface have also been shown to influence keel bone deformations (16). Keel damage in other low-impact systems, namely, conventional cages equipped with perches, also reported increased keel damage with the perch provision (15). The greater amount of perch use in fractured hens reported in this study likely parallels this causal hypothesis that increased exposure to continuous loading pressure on the keel causes damage. This is further supported by the behavioral choice of hens with intact keels to rest on the floor of the cage more often than on the perch while fractured hens spent significantly more time resting on perches, with their keel in contact with the perch surface, than their intact counterparts. The long-term pressure of the perch on the keel during sitting or sleeping behavior may be causing the deformation and fractures. Although this theory of perch use causing fractures in furnished cages correlates with previous research, within this study is it impossible to definitely support this causal hypothesis since hen selection criteria was based on palpation for keel status and perch use behavior prior to keel damage is unknown.

Alternatively, it is possible that this difference in perching behavior could be a coping strategy to use the perch as a support structure either to relieve strain on the keel by relaxing the attached abdominal muscles or to reduce the involvement of the keel in respiration. This concept also applies to standing behavior, which requires substantial oscillatory movement of the keel during respiration in a standing position and increases the strain and pressure on the keel due to the added gravitational weight of the visceral organs.

In domestic fowl, Hocking et al. reported that sitting and standing behaviors are influenced by both naturally induced pain and pharmacologically induced pain models and the behaviors are subsequently restored with administration of analgesics (8) and anti-inflammatory steroids (11). These behavioral changes are suggested to be an attempt to reduce weight bearing on the injured limb or relieve pressure on the spine. Not only this but also standing is costly, requiring a 40–45% increase in metabolic effort (31) and causing 20–40% greater heat loss (32) when compared to sitting. Although the keel bone is not a load bearing bone for standing, it is a bone that is subjected to the gravitational weight of the internal organs. When the keel is resting on a supportive surface, the degree of involvement of the attached abdominal muscles to support the visceral weight is decreased. Therefore, it is possible that in a strategy similar to reducing load bearing on painful limbs, the reduction of standing behavior may be a mechanism of pain relief for hens with fractured keels by limiting the time spent in an metabolically costly position where the visceral weight adds to the strain on the keel induced by the activation of the external oblique. This hypothesis requires further research involving the administration of analgesics.

In a similar manner, this increase in perching and reduction in standing behavior may be an attempt to alter physiological function in relation to respiration. The keel plays a vital role in driving the respiratory process; however, its involvement and displacement during inhalation and exhalation changes when the hen is in a sitting vs. standing position. When the hen is seated in a resting position with the keel flush against a surface, such as the floor or perch, the oscillatory movement of the keel is restricted, and subsequently the involvement of the ribs becomes more substantial (19, 20). When the keel is restricted, the appendicocostalis drives the expansion of the thoracic cavity by flaring the ribs laterally, whereas when standing, the keel is allowed full oscillatory movement with the external oblique primarily responsible for pulling the keel dorsally during the exhalation (19). When the keel is fractured, the hen could be subjected to reduced flexibility or mobility of the keel and its muscle attachments forcing the involvement of alternative respiratory strategies, or the hen could be intentionally reducing the involvement of the keel in respiration by reducing standing behavior and increasing the time spent supporting the keel on the perch. Since there was no significant difference in sitting duration in regard to fracture severity, it cannot be assumed that the hens are directly substituting standing behavior with sitting; however, the reduction in stationary standing suggests that aspects of this position are possibly uncomfortable or less efficient. This hypothesis is supported by the shorter bout duration for standing and the increased number of sitting bouts expressed by hens with keel fractures. Perhaps, the desire to rest is present, but discomfort or metabolic inefficiency induces a more restless expression of behavior.

The proportion of the behaviors reported here are similar to previous descriptions of general activities including dustbathing (25, 28), preening (25, 26, 33), sitting (33), walking, resting (25), sleeping, eating, and drinking (26). Foraging was noticeably lower in this study compared to previous reports in red jungle fowl (33) and LSL hens (25); however, this is likely due to genetic differences between red jungle fowl and modern lines in terms of the degree of time budget devotion to energetically costly activities (34, 35). It is also possible that the classification of eating and foraging in the ethogram used here led to mixing of the two behaviors, since LSL hens are known to spend more time feeding and foraging in the feed trough rather than scratching at the floor (25). The differences in the percentage of time standing reported here is likely a consequence of housing. Caged hens spend a large portion of their time standing at 70–75% of their daily time budget (26), whereas hens in natural environments and floor pens reduced the time standing to as low as 5% in red jungle fowl (33) and 10–12% in LSL hens (25). Furnished cages likely lie in between conventional cages and floor pens in terms of the range of expression of behaviors as they offer opportunities for a greater diversity of activities than conventional cages; yet, the complexity of the environment and overall space allowance is typically lower in furnished cages than in natural or floor pens.

Although not of primary concern, the effects of several covariates on behavior add to the discussion and provide evidence that the focal sampling technique used here adequately captured behavioral patterns. The effect of rearing environment and cage size on perch use reported here are likely related to different learned behaviors and use of space. Chicks and pullets offered perches early in life have been shown to maintain different spatial usage later in life compared to pullets without early perch access (36–38). In addition, even though the individual space allowance in both the large and small cages were equal, the overall increased space to navigate and larger nest area in large cages has the potential to alter the movement and flow within the cage design. The inverse relationship between body weight and the proportion of both foraging and walking behavior is not surprising, and neither is the increased foraging on the top tier which was noticeably brighter due to its closer proximity to the light source compared to the middle and lower tiers.

One final question to address is why hens with minor fractures more closely resemble the behavioral description of hens without keel fractures than hens with severe keel damage. The lack of behavioral difference between *F*_0_ and *F*_1_ hens with fractures at the caudal tip begins to address the question of whether or not this minor damage to the keel is negatively impacting the hen. From the results presented here, it appears that minor caudal tip fractures do not significantly impact the daily activity of the hen in the same way that keels with large deviations and multiple fractures sites do. This may be in large part due to the type of fracture occurring in each case.

Unlike the complete, displaced fractures seen in severely fractured keels, classified by complete separation of the bone and displacement from its original position, the minor caudal tip fractures appear to more closely resemble a “greenstick” fracture. Greenstick fractures are characterized by an incomplete fracture on the concave side of a bone and a complete separation on the cortex on the convex region of the bone (39, 40). The minor caudal tip fractures reported in this study followed this description with a small callus indicative of a healing fracture on the convex tip of the keel and evidence of bending on the concave portion of the caudal tip. In a previous report using an LSL-Lite flock raised at the same research station as this study, this type of fracture was reported to be responsible for 64% of the total fractures present (22). It is possible that this minor fracture, although still potentially painful, does not disrupt the periosteal nociceptors enough to dramatically alter behavior.

One concern with greenstick fractures is that they are considered unstable and are at risk of further displacement for several weeks after the initial injury (41). Even though the *F*_1_ hens spent a lower total proportion of time on the perches than *F*_2_ hens, almost half of their time on the perches was for resting, closely resembling the *F*_2_ group. While this mixed result is possibly related to a small sample size and larger standard deviation for the *F*_1_ group in terms of resting location, it could also be relating back to a causal relationship between perch use and fractures suggesting hens with greenstick fractures are at risk for more extensive, fracture displacement injuries in the future as a result of their time spent resting on the perches.

Since the primary goal of this study was to assess behavioral expression and resting location in relation to keel bone damage in furnished cages, it is now possible for further studies to be designed to quantify the behavioral differences reported here in more detail and potentially assess changes to these behaviors with administration of analgesics.

## Conclusion and Animal Welfare Implications

Keel bone damage is a welfare concern in all housing systems. Understanding how keel bone fractures are caused by certain behaviors or how they subsequently alter behavior can provide valuable information for appropriate housing design, genetic selection traits, and identification of affected hens in commercial settings. In addition to further research into pain related to keel bone damage, identifying physiological and metabolic effects of severe keel bone damage will add to the scope of understanding of this prevalent welfare concern.

## Author Contributions

TC-T designed, conducted, and analyzed the data for this report, as well as wrote the manuscript for submission. TW contributed to the design and analysis of the data, as well as edited and supplemented the written manuscript. Both authors contributed to planning the study, writing the manuscript, and approved the final version.

## Conflict of Interest Statement

The authors declare that the research was conducted in the absence of any commercial or financial relationships that could be construed as a potential conflict of interest.
